# Evaluation of Graded Levels of Fermented Plant Protein (Proteger^®^) on Extrusion Processing and Diet Utilization in Young Cats

**DOI:** 10.3390/ani15070918

**Published:** 2025-03-22

**Authors:** Youhan Chen, Charles Gregory Aldrich

**Affiliations:** Department of Grain Science and Industry, Kansas State University, Manhattan, KS 66506, USA; youhan23@ksu.edu

**Keywords:** soybean protein, bioprocess, cats, extrusion, digestibility, stool quality, palatability, hind-gut fermentation

## Abstract

This study evaluated fermented plant protein (FPP) for extrusion performance and its effects on diet utilization in adult cats. Four diets were formulated: a control with 15% soybean meal (SBM) and three diets replacing soybean meal at 5%, 10%, and 15% with FPP (5FPP, 10FPP, and 15FPP). Diets were extruded using a single-screw extruder, with data and samples collected at 15 min intervals. Twelve cats participated in a 4 × 4 Latin square design, with 9 days of diet adaptation followed by 5 days of total fecal collection for nutrient digestibility and colonic fermentation analysis. Processing effects of FPP inclusion were minimal. The kibble sectional expansion index was lowest for SBM (2.50) and highest for 15FPP (2.82; *p* < 0.05). Fecal moisture increased (*p* < 0.05) in cats fed 15FPP. The apparent total tract digestibility of protein was greatest (*p* < 0.05) in cats fed 15FPP. The fecal ammonia concentrations increased linearly (*p* < 0.05) with FPP, while total fatty acid concentrations were lower (*p* < 0.05) in cats fed 10FPP compared to SBM. Cats preferred (*p* < 0.05) 10FPP over SBM as measured by the intake ratio. The FPP inclusion (up to 15%) did not adversely affect processing, stool quality, or nutrient digestibility. Diets with 5–10% FPP tended to reduce hind-gut fermentation.

## Simple Summary

Soybean meal is a common plant-based protein ingredient in extruded pet foods. However, a high inclusion level of soybean meal in pet food is limited by antinutritional factors, including trypsin inhibitors and oligosaccharides, that compromise protein digestibility, stool quality and cause flatulence in cats. Our animal study found that inclusion of a fermented plant protein product at 10% in a cat diet promoted kibble expansion; and had comparable protein digestibility, less hind-gut fermentation and greater palatability with ideal stool quality in cats when replacing traditional soybean meal by 10 out of 15% of the formula. We suggest that fermentation with Aurobasidium pullulans was an effective method to improve soybean meal nutritional value, hence increasing the inclusion level in extruded cat foods.

## 1. Introduction

Cats, as strict carnivores, have protein requirements that are 2 to 3 times higher than other non-carnivores for adult maintenance due to the inability to downregulate nitrogen loss through the urea cycle [1,2]. Cat owners prefer animal-based proteins to plant-based proteins, for the former provide a better balance of essential amino acids for cats [3]. It has been forecasted that by 2030, 35% of USA households will own a cat [4]. Production of complete and balanced commercial cat foods demands a large volume of protein, but this may have negative effects on sustainability due to animal slaughter, which is a concern to portions of the public [5]. Offsetting some of the animal-based proteins with vegetable sources might alleviate some of this concern for long-term sustainability, presuming that the vegetable-based proteins contain appropriate levels of bioavailable essential amino acids.

Soybean meal is a complementary plant protein source, produced by grinding soybean cake after oil extraction [6]. Plant proteins are incomplete proteins to cats as feeding them alone will not meet the animals’ requirement for amino acids. Most plant proteins are limited by lysine content for cats while legumes such as soybean are limited by methionine [7,8]. Such characteristic endows soybean protein the capability to complement with other plan proteins to formulate nutritionally complete cat diets. However, inherent antinutritional factors compromise the nutritional value of soybean meal, such as trypsin-inhibitors decreasing protein digestibility and oligosaccharides causing flatulence and poor stool quality [9,10,11]. Apart from that, traditional soybean meal is generally not appreciated by pet owners as an ingredient as it is considered to be inferior with a bias in marketing “no soy” in some premium branded products. Reasons include a biased perception of soybean protein allergenicity, relatively poor protein quality, genetically modified organisms, poor digestibility, antinutritional factors and other health/nutrition related concerns [12,13].

To promote the utilization of soybean meal in cat foods, we need to improve its customer impression and nutritional value. Fermentation may be helpful for both. There exists production of fermented plant protein (FPP) through enzyme-assisted fermentation of soybean meal [14] that has proven to be superior compared to the unfermented ones in farm animal species and it may overcome some of the pet food consumer acceptability issues since fermentation is generally considered beneficial for food processing [15]. Inclusion of FPP in feed has been shown to enhance nutrient digestibility and overall feed efficiency in weaned piglets and calves [16,17]. This same response could make it an attractive ingredient for pet food formulations. However, information in this regard is not readily available as it pertains to the potential impacts of FPP or any other fermented soybean products on stool quality, diet digestibility, and intestinal health when fed to cats. Our hypothesis is that the addition of FPP in extruded food will not have negative effects on stool quality, nutrient digestibility and palatability in cats.

## 2. Materials and Methods

### 2.1. Diet Formulation and Production

The FPP used in this research is a commercial product derived from genetically modified soybean meal through a proprietary fermentation process (Houdek Manufacturing, Volga, SD, USA). Four cat diets were formulated to be nutritionally balanced for adult cats according to AAFCO (2016) [18] (Table 1). The soybean meal in the control diet (SBM) was replaced by FPP at 5, 10, and 15% (5FPP, 10FPP, and 15FPP, respectively). The experimental cat diets were formulated to have high protein (>35%) and a moderate level of fat (>12%). Chicken meal and low ash chicken meal were used to balance ash content as soybean meal replaced by FPP. Titanium dioxide (TiO_2_; 0.4%) was included in each diet as an indigestible marker for determination of the apparent total tract digestibility (ATTD) of dietary nutrients. As predicted by the formulation software (Concept 5^©^, CFC Tech Services, Inc., Staples, MN, USA), the concentrations of minerals (calcium, phosphorous, potassium, magnesium, sodium, sulfur, manganese, copper iron, and zinc) were similar among diets and met AAFCO nutrient profile recommendations for adult cats at maintenance.

The dry expanded pet foods were produced using a single screw extruder (model X115, Wenger Manufacturing, Sabetha, KS, USA). The preconditioner (PC; model HIP 150, Wenger Manufacturing, Sabetha, KS, USA) was operated at a constant speed (20% mix intensity) with welded paddles. The extruder had a defined profile and barrel temperatures based on a typical commercial pet food configuration. At the end of the extruder barrel, there were 2 inserts of 10 openings (5.00 mm diameter) per insert. Fixed input parameters were kept constant throughout each individual food production and included dry blends feed rate (596.8 ± 5.1 kg/h), PC water (118 ± 1.0 kg/h), PC steam (30.0 kg/h) and extruder screw speed (350 rpm).

During the extrusion process, the PC and extruder output parameters were all collected from sensor readouts every 15 min to estimate the effects of different inclusion levels of FPP on extrusion. Output variables were those parameters resulting from the input variables, and included extruder die temperature, extruder motor load and specific mechanical energy (SME). Measurements were collected every 15 min during the production for a total of at least 2000 lb per experimental diet and were considered treatment replicates.

Wet extrudates were conveyed pneumatically through an air hood system and deposited onto an oscillating belt spreader that spread the kibble evenly across the dryer bed. The kibble were dried on a belt passing two zones of a three-zone dryer (Airflow II, Wenger Manufacturing, Sabetha, KS, USA) to achieve the moisture content of kibble below 10%. The dryer zones had varied temperatures and retention time as 19 min in zone 1, 10 min in zone 2 and 29 min in zone 3. Dried kibbles were coated with chicken fat and dry digest (Manx, AFB International, St. Charles, MO, USA) in a drum mixer. Coated kibbles were stored in room temperature in poly-lined paper bags until fed.

Specific mechanical energy (SME) was calculated using the following formula:

$$\mathrm{SME}\;(\mathrm{kJ}/\mathrm{kg})=\frac{\frac{\left(\tau -\tau_{0}\right)}{100}\times \frac{N}{\;N_{r}}\times P_{r}}{m}$$

where τ is the extruder motor load, τ_0_ is the extruder no load % torque, N is the extruder screw speed (rpm), N_r_ is the rated extruder screw speed, and P_r_ is the rated extruder motor power, and m is dry feed rate (kg/s).

The kibble diameter and thickness (length) of 10 randomly selected kibbles from each collection point of each diet production off the extruder and off the dryer were measured with a digital caliper. The sectional expansion index (SEI) was determined by comparing the squared diameter of the dried extruded kibble by the squared die diameter of the extruder:

$$\mathrm{SEI}=\frac{D^{2}}{d^{2}}$$

where D (mm) is the extrudate diameter and d (mm) is the extruder die diameter.

Off the extruder kibble bulk density was measured manually off the extruder in each data collection point during each treatment processing using a 1 L cup and leveling the kibble with a metal ruler and weighing on a digital scale with 1.0 g sensitivity.

### 2.2. The Feeding Trial

Twelve healthy American shorthair cats (6 males and 6 female) of similar age (10 month ± 5 days) were enrolled in this study. The cats were designated research animals housed at Coles Hall at Kansas State University (same location where this feeding trial was conducted). They had an average body weight of 4.6 ± 1.4 kg, and food allowance was controlled to maintain their weight throughout this study. The daily metabolizable energy (ME) requirement was calculated for laboratory cats [100 × BW, kg^0.67^ (NRC, 2006) [19]]. The cats were housed on a 12 h light cycle with lights off from 1900 to 0700. In the adaption period, the cats were group-housed but fed individually. Whereas in the collection period, the cats were individually housed in stainless steel cages. The cats received two feedings per day at 0800 and 1600 h with access to food for 1 h in equal ration to meet daily food allowance. In case a cat refused to eat an experimental diet, an additional 0.5% to 1.0% flavor enhancer was added topically to the food.

The ME of experimental diets were calculated using the prediction equation in pet food [(8.5 × CF) + (3.5 × CP) + (3.5 × NFE)]. The daily food allowance was calculated using the daily ME requirement divided by predicted ME of diets. The body weight of each cat was measured at the beginning, middle, and end of each period and their food allowance was adjusted by 5 or 10% for the following week to maintain their BW. Water was provided for ad libitum consumption.

The feeding trial used a replicated Latin square design, where each cat served as its own control. Each of the four periods composed of 9-day for adaptation followed by 5-day of collection. During collection period, all feces and orts were collected daily. Every fecal sample was weighed and scored on a 5-point scale, where 1 = liquid diarrhea; 2 = very soft consistency, unformed stool; 3 = soft stool that retains shape; 4 = well-formed firm stool that does not leave residue when picked up; and 5 = very hard, dry stool [20]. Fecal samples were stored at −20 °C until the end of trial and then thawed at room temperature, pooled by cat, weighed, and dried in a forced air oven at 5 5°C for up to 48 h until the moisture level was below 10%. The partially dried fecal samples were also weighed, and the values were used when calculating the DM (dry matter) of the fecal samples. Diet samples and partially dried fecal samples were ground through a 1 mm screen by a laboratory fixed blade impact mill (Retsch, type ZM200, Haan, Germany) and stored in Mason jars at room temperature for further chemical analysis.

In addition, the pH of one fresh fecal sample per cat per period (within 15 min of defecation) was recorded in triplicate with a calibrated glass-electrode pH probe (FC240B, Hanna Instruments, Smithfield, RI, USA) immediately after collection. Fresh fecal samples were stored at −80 °C until further analysis.

### 2.3. Chemical Analysis

The amino acid composition of FPP sample was analyzed at the University of Missouri Agricultural Experiment Station Chemical Laboratories (Columbia, MO, USA; AOAC 982.30 and 988.15). All amino acids, except methionine, cysteine, and tryptophan, were digested with 6 N HCl for 24 h at 110 °C. The amino acids were then separated by ion-exchange chromatography and the concentration was determined with a Beckman 6300 amino acid analyzer (Beckman, Palo Alto, CA, USA). Methionine and cysteine were first oxidized by performic acid to methionine sulfone and cysteic acid, respectively, prior to acid hydrolysis. Tryptophan was hydrolyzed in 3 M mercaptoethanesulfonic acid before analysis. Available lysine was determined (AOAC 975.44) and lysine availability (%) was calculated as the ratio of available lysine to total lysine. Amino acid contents of the FPP were used to calculate protein corrected amino acid scores (PDCAAS) using the Food and Agricultural Organization suggested equation [21]:

$$\mathrm{Amino}\;\mathrm{acid}\;\mathrm{score}\;(\mathrm{AAS})=\frac{\left(\mathrm{mg}\;\mathrm{of}\;\mathrm{amino}\;\mathrm{acid}\;\mathrm{per}\;1\;g\;\mathrm{crude}\;\mathrm{protein}\right)}{\left(\mathrm{mg}\;\mathrm{of}\;\mathrm{same}\;\mathrm{amino}\;\mathrm{acid}\;\mathrm{per}\;1\;g\;\mathrm{crude}\;\mathrm{protein}\;\mathrm{requirement}\right)}$$

PDCAAS = AAS of LAA × CP digestibility

The ground experimental diet samples (after coating) and fecal samples were analyzed for dry matter (DM), organic matter (OM), and ash according to methods in AOAC 934.01 and 942.05. Crude protein was determined (AOAC 990.03) using a nitrogen analyzer (FP928, LECO Corporation, Saint Joseph, MI, USA). Crude fat was determined by acid hydrolysis followed by hexane extraction (ISO 11085:2008) [22] using semi-automated equipment (Hydrotec 8000 and ST 255 Soxtec, Foss, Denmark). Gross energy was measured as the total heat of sample combustion by calorimetry (Parr 6200 Calorimeter, Parr Instrument Company, Moline, IL, USA). The titanium dioxide content in diet and fecal samples was analyzed with a colorimetric method [23]. The soluble dietary fiber (SDF), insoluble dietary fiber (IDF) and total dietary fiber (TDF) content of diet samples were measured (AOAC 991.43) by an ANKOM Dietary Fiber Analyzer (ANKOM Technology, Macedon, NY, USA).

The fecal ammonia concentration in the fresh fecal samples was analyzed through colorimetric method described by Chaney and Marbach (1962) [24]. The fresh fecal samples were thawed and diluted with deionized water and 0.1 N HCl and homogenized. The homogenized samples were centrifuged at 3000× *g* for 20 min to separate the suspended solids. The 1 mL of the supernatant of the centrifuged samples was collected and kept frozen at −20 °C for at least 24 h to complete deproteinization. The acidified samples were thawed, centrifuged at 20,000× *g* for 15 min, and plated.

Fecal short-chain fatty acids (SCFAs) and branched-chain fatty acids (BCFAs) contents were analyzed on a gas chromatography (GC; Agilent 7890, Agilent Technologies, Santa Clara, CA, USA). The fresh fecal samples were thawed and diluted with deionized water and homogenized, followed by centrifugation at 3000× *g* for 20 min to separate the suspended solids. The 1 mL of the supernatant of the centrifuged samples was collected acidified with 0.25 mL of 25% m-phosphoric acid was before deproteinization at −20 °C for at least 24 h. The GC is equipped with flame ionization detector and a capillary column (DB-WAX, Agilent 127-7012, 10 m × 0.1 mm × 0.1 µm, Agilent Technologies, Santa Clara, CA, USA). Hydrogen was used as a carrier gas with a flow rate of 35 mL/min, and the split ratio was 100:1 with injection volume of 0.2 µL. Nitrogen was used as the makeup gas with a flow rate of 25 mL/min. The detector and injector temperatures were set at 300 °C, and the initial oven temperature was set to 40 °C with a ramp rate of 20 °C/min to 180 °C for a total run time of 8 min. The peak area of chromatograms was determined using an integrative software (OpenLab CDS version 2.8, Agilent Technologies, Santa Clara, CA, USA). The concentrations of SCFAs (acetate, propionate, and butyrate) and BCFAs (isobutyrate, isovalerate, and valerate) in samples were quantified by comparing the sample peak area to three standards with known concentrations of each volatile free acid and correcting for the fecal dry matter content.

### 2.4. Digestibility Calculation

Two methods were utilized to estimate apparent total tract nutrient digestibility. The total fecal collection (TFC) method requires the collection of all feces excreted by the experimental animals. The marker method uses TiO_2_ as an indigestible dietary marker. In the current study, the ATTD of DM, OM, CP, CF and GE were calculated according to the TFC and marker methods by following equations:

The TFC method:

$$Nutrient\;digestibility\;\left(\%\right)=\frac{\left(nutrient\;consumed\;\left(g\right)-nutrient\;excreted\;\left(g\right)\right)}{nutrient\;consumed\;\left(g\right)}\times 100\%$$

The marker method:

$$Nubrient\;digestibility\;\left(\%\right)=[1-\frac{\left(nutrient\;in\;feces\;\%\;\times TiO2\;in\;food\;\%\right)}{\left(nutrient\;in\;food\;\%\;\times TiO2\;in\;feces\;\%\right)}]\times 100\%$$

### 2.5. Palatability Trials

The FPP-containing diets (5FPP, 10FPP and 15FPP) were evaluated for palatability versus the non-FPP diet (SBM) by a cat panel, different from those in the feeding trial above, at a commercial facility (Summit Ridge Farms, Susquehanna, PA, USA). Thirty domestic short hair cats (15 males and 15 females, all neutered) were of differing ages (1.5–12.5, averagely 6.8 ± 3.12 years-old) while all adapted towards eating various food formats. Each experiment was conducted as a split-plate test, in which two stainless steel bowls containing 100 g of food were presented to animals for a total of 4 h. Each comparison trial was repeated for 2 d, with bowl position switched daily. Thirty cats were fed daily, providing 60 observations for each paired comparison test. Preference was determined based on animals’ first choice and total food consumption. Data from consumption were represented as the following ratio:

$$\mathrm{Intake}\;\mathrm{ratio}=\frac{consumption\;of\;Diet\;A}{consumption\;of\;Diet\;A+Diet\;B}\times 100\%$$

### 2.6. Statistical Analysis

Least square means of data were estimated by a general linear mixed model in a software (GLIMMIX, SAS version 9.4, SAS Institute Inc., Cary, NC, USA). Pairwise comparisons were conducted using Tukey’s post hoc test. Contrasts comparing control (SBM) versus treatments (5FPP, 10FPP, and 15FPP), and linear, quadratic, and cubic relationships among all diets were considered significant at *p* < 0.05. For each diet production, sampling was conducted at evenly spaced intervals which were considered replicates. For digestibility trial analysis, the diet was considered a fixed effect while the cat and period were considered random effects in the analysis model. In the palatability experiment, the consumption ratio was analyzed using a t-test in a two-way ANOVA, and the first-choice preference was analyzed using a Chi^2^ test.

## 3. Results

### 3.1. Chemical Analysis of FPP and Diets

Total essential amino acids comprised 45.5% of total crude protein in FPP with (phenylalanine + tyrosine) serve as the limiting amino acid(s) for cats (Table 2) based on the NRC recommended allowance of essential amino acids and crude protein [19]. All diets contained an average CP concentration of 37.0% (35.6–37.9%), an average fat concentration of 13.3% (12.9–13.7%), an average TDF concentration of 17.2% (15.8–20.2%), and an average GE content of 4861 kcal/kg (4686–4999 kcal/kg) on DM basis (Table 1). However, the SBM had high TDF and IDF content compared to FPP-containing diets.

### 3.2. The Extrusion Process

Input parameters remained similar among diets except knife speed that was manually decreased for 10FPP and 15FPP compared to SBM and 5FPP (Table 3). A cubic relationship (*p* < 0.05) between PC steam and FPP inclusion was observed. A linear relationship between knife speed and FPP inclusion was observed (*p* < 0.001). The diameter, thickness and SEI of extrudates increased linearly (*p* < 0.001) as FPP inclusion increased. The extrudates of FPP-containing diets were more expanded (*p* < 0.001) than that of SBM.

### 3.3. Feed Intake and Fecal Characteristics

Food intake, and wet and dry fecal output remained similar among cats fed with experimental diets. Fecal moisture content in cats fed 15FPP (69.78%) was greater (*p* < 0.05) than that of cats fed with other experimental diets (67.70%, 67.04% and 67.12% for SBM, 5FPP and 15FPP) and showed a cubic relationship (*p* < 0.001) with FPP inclusion (Table 4). Fecal score and defecation frequency (at an average of 3.85 and 1.1 time/day, respectively) of cats were also not affected by dietary inclusion of FPP.

### 3.4. Apparent Total Tract Digestibility

The ATTD of nutrients calculated with both the TFC and the marker method (TiO_2_) had similar standard error of mean (SEM) values and generally agreed with each other in regard to rankings (Table 5). Although statistically significant differences in the ATTD were more frequent with the marker method. With the TFC method, the ATTD of dry matter, organic matter, crude protein and gross energy were not different among cats fed with experimental diets. The ATTD of crude fat of cats fed with 5FPP (92.17%) was lower (*p* < 0.05) than that of cats fed with diets with 10% and 15% FPP (94.56% and 94.98%, respectively). A quadratic, a linear and a cubic model was found (*p* < 0.05) in the predicted ATTD of crude protein, gross energy and crude fat, respectively, in cats fed with diets with varied FPP inclusion levels. On the other hand, the ATTD of dry matter, organic matter, crude protein and gross energy calculated with the titanium dioxide marker method showed cubic relationships (*p* < 0.05) with FPP inclusion (Table 5). The ATTD of crude protein was the greater in cats fed with 15FPP (72.5%) than that of cats fed with SBM and 10FPP (67.3% and 67.8%., respectively). There was a cubic relationship (*p* < 0.05) between the ATTD of crude fat and FPP inclusion, but cats fed with 0% FPP (91.3%) had an intermediate crude fat digestibility among cats fed all experimental cat diets (from 89.7 to 93.1%).

### 3.5. Colonic Fermentation

Though fecal pH did not differ among cats fed with different diets, the ammonia concentration in fresh feces increased linearly (*p* < 0.05) as FPP level increased in diets (Table 6). The concentrations of acetate, propionate, valerate, total SCFA, total BCFA and total fatty acids had a cubic relationship (*p* < 0.05) with FPP inclusion. The total SCFA concentration was lower (*p* < 0.05) in feces from cats fed with the 10FPP (384.9 μmol/g DM feces) than that from cats fed with the SBM (452.8 μmol/g DM feces). Cats fed with 10FPP (407.1 μmol/g DM feces) also had the lowest (*p* < 0.05) total fatty acid concentrations in fresh feces compared to cats fed with SBM and 15FPP (480.1 and 483.6 μmol/g DM feces, respectively).

### 3.6. Palatability

Cats showed preference (*p* < 0.05) for 5FPP to SBM on the first day according to first choice, but such preference did not last (Table 7). The intake ratio did not show any difference between SBM and 5FPP. When comparing SBM and 10FPP, cats showed preference (*p* < 0.05) for 10FPP indicated by the two-day intake ratio but not first choice. For 15FPP, cats only showed preference (*p* < 0.05) for it over SBM on the second day indicated by the intake ratio.

## 4. Discussion

### 4.1. Chemical Analysis of FPP and Diets

In the past, cats were a much smaller proportion of the pet population compared to dogs and were not considered as a direct model for human nutrition/medicine since they are strict carnivore. Therefore, fewer research publications have been available regarding cat nutrition, foods and various ingredients used therein. With a shorter small intestine relative to body weight for cats compared to dogs, one would predict a lower apparent digestibility of AAs for lower-quality proteins; but similar values for high-quality proteins (e.g., those with 90% or higher apparent digestibility) [25]. The FPP has lower protein/peptide molecular weights compared to regular soybean meal protein, which led to higher digestibility in terrestrial animals and was verified through in vitro protein digestibility (98.5%) in this study. The sulfur-containing amino acids (SAA, methionine and cysteine) are usually the limiting amino acids in legumes for cats. However, the AAS for SAA in FPP was 2.43. Even the AAS for methionine alone was 1.62, suggesting that the FPP is a legume-originated plant protein source with distinctive amino acid profile compared to other plant proteins. Cats also have a distinctive requirement for arginine to prevent ammonia intoxication [26]. The FPP had an AAS for arginine greater than 1.0 for cats even after taking protein digestibility into account. Phenylalanine plus tyrosine are responsible for maintaining black hair color of cats [27]. Thought the AAS for phenylalanine plus tyrosine in FPP was the lowest among all essential amino acids, it was still over 1.0 with protein digestibility accounted. Additionally, there was a low level of taurine, a non-proteinogenic but essential dietary nutrient for cats [1], found in measurable quantities in FPP(0.41 g/mg CP) which was surprising since this amino-sulfone is not typically associated with plant-based proteins. This reflects the fungal biomass’s contribution to amino acid composition in the FPP, as taurine has been detected in some species [28]. Based on these factors FPP appears to be a reasonable quality plant protein for consideration in cat diets if formulated as a complement to animal proteins or with judicious use of purified amino acid additives.

The experimental diets produced in this study met the targeted moisture content of approximately 5% (4.28–6.27%) well below the 10% threshold that might be considered to permit mold growth [29]. The slight increase in CP content from SBM to FPP-containing diets was anticipated based on the analysis of the experimental sample of FPP (77.03% DM basis) which had a much higher protein level than the soybean meal (53.8% DM basis) used. The decrease in CP in 15FPP compared to the other two FPP-containing diets was most likely due to sampling or analytical variation. Low ash content in 15FPP was also anticipated as it had the most low-ash chicken meal included. Considering that fiber sources in all experiment diets were similar except soybean meal and FPP, the decreased TDF and IDF in FPP-containing diets indicated that the FPP unexpectedly had much lower content of them compared to the soybean meal used in this study. The similar SDF among diets reflected less difference in SDF than in IDF between FPP and soybean meal used in this diet. The slight increase in SDF in 5FPP was possibly due to analytical variation. The differences in dietary fiber contents between this fermented soybean meal product and regular soybean meal provides valuable information when formulating diets with them in the future, especially when hind-gut fermentation is of interest.

### 4.2. The Extrusion Process

Generally speaking, replacement of soybean meal with FPP in this study up to 15% did not lead to drastic changes in extrusion processing conditions for proper extrudate expansion or bulk density. The SBM had the least kibble expansion versus the FPP-containing diets; possibly due to the high IDF content as inclusion of IDF usually decreases sectional expansion [30,31]. The increased longitudinal expansion of SBM kibble compared to that of 5FPP kibble were consistent with other studies in which it was observed that decreased sectional expansion occurs when increasing IDF content often leads to increased longitudinal expansion [32,33]. The increased kibble thickness in 10FPP and 15FPP were expected as the knife speed was manually decreased during production. Before adjustment of knife speed, the kibble from these two diets were too thin indicating decreased longitudinal expansion. This agreed with previous observations of negative relationship between sectional and longitudinal expansion [32,33]. The possible mechanisms are decreased sectional elastic recovery at the die exit facilitated energy impact on increased longitudinal expansion [34] and addition of fiber as nucleation agents decreased energy requirement for bubble nucleation and led to earlier nucleation in the die and favored stretching of the bubbles in the direction of the melt flow, thus greater longitudinal expansion [35]. If we compare dog diets with FPP, the cat kibble had greater SME and less expansion compared to dog kibble, which can be explained by less starch in cat diets to create viscosity and elasticity. In summary, the replacement of soybean meal with FPP at up to 15% promoted expansion and did not pose any challenges in extrusion techniques. The contribution to expansion seemed to derive from the great dietary fiber composition between soybean meal and FPP used in this study.

### 4.3. Feed Intake and Fecal Characteristics

Energy is the major driving factor for food intake of cats and dogs [36]. The similar food intake was expected as all diets had similar gross energy content. Though statistically different, the increase in fecal moisture content in cats fed with 15FPP was of limited practical influence as fecal scores and wet fecal output were maintained among cats fed with each experimental diet. However, the fecal wet and dry matter output were numerically lower in cats fed with FPP-containing diets compared to that of cats fed with the non-FPP diet. This may lead to an increase in nutrient digestibility with inclusion of FPP (later in this article). It has been reported that IDF decreased apparent digestibility and increased fecal dry matter content and fecal bulk in monogastric animals [37,38]. The SBM diet had more IDF than other experimental diets, which could explain the higher fecal dry matter percentage/lower moisture percentage in feces from cats fed with the SBM.

### 4.4. Apparent Total Tract Digestibility

As mentioned previously, the increased ATTD of dry matter in cats fed with the 15FPP was consistent with the lower fecal dry matter content compared to that of cats fed with the other three diets. The ATTD of all nutrients in cats fed with FPP-containing diets were comparable or even greater than those of cats fed with the control diet. Such results can be attributed to three major aspects: differing dietary fiber content among diets, higher protein digestibility of FPP and lower oligosaccharides in FPP. The high IDF and TDF content in the control diet compared to FPP-containing diets may have impacted nutrient digestibility. Previous studies have reported similar decreases in nutrient digestibility in cats as fiber levels increased [39,40,41]. The mechanisms by which fiber decreases nutrient digestibility include shorter gastrointestinal tract transit time, nutrient binding, physical blockage to enzyme access, increased endogenous losses and reduction in brush border enzyme activity [42,43,44]. The high protein digestibility is one of major advantages in FPP since fungal fermentation and enzyme addition have degraded large protein molecules into smaller peptides to facilitate digestion [14]. The FPP is free of soybean oligosaccharides (OS) that are considered an antinutritional factor. Highly fermentable soybean OS may decrease nutrient digestibility by stimulating faster transition and forming complexes with other nutrients [45]. Low-OS low-phytate soybean meal was found to increase the ATTD of dry matter in dogs compared to regular soybean meal [46]. Similarly, the FPP was reported to increase the standardized ileal digestibility of protein and some amino acids in post-weaning piglets when compared to fish meal [14].

However, the ATTD of crude protein in this study seemed to be low regardless of treatments. Two previous cat studies evaluating corn fermented protein with similar study design (most importantly, using 15% soybean meal in the control diets) to ours both found the ATTDs of CP above 85% (calculated by the TiO_2_ marker method) [40,47]. The differences in dietary fiber compositions did not seem to explain such discrepancy because the ATTD of other macro-nutrients were comparable among all three studies. Cats used in this study were approximately 10 months old which should be more than adequate given that the protein digestion capacity reaches adult level as early as in 19-week old [48]. Processing might have played a role in the relatively low protein digestibility in this study. Studies suggest that high SME levels generally improve digestibility by breaking down complex protein structures, making them more accessible for digestive enzymes [49,50]. The SME in this study was notably lower than commonly seen in extruded pet food [30,47,49,51].

### 4.5. Colonic Fermentation

The SCFAs are organic fatty acids with 1 to 6 carbon atoms [52], resulting from the incomplete fermentative metabolism of carbon-containing molecules. However, to differentiate fatty acids from carbohydrate fermentation and protein fermentation in this study, SCFAs specifically relate to acetate, propionate and butyrate while BCFAs refer to isobutyrate, valerate and isovalerate. Colonic fermentation in dogs and cats plays a crucial role in maintaining gastrointestinal health by breaking down undigested carbohydrates into SCFAs, which decrease fecal pH, provide metabolizable energy for colonic cells and promote overall gut health [53]. Undigested proteins and peptides also can be substrates for colonic bacteria. The deamination and decarboxylation of amino acids produce a complex of protein fermentation products such as ammonia, BCFAs (e.g., isobutyrate and isovalerate), indoles, phenols and volatile sulfur-containing compounds [54]. Many of these compounds are found to have adverse impacts on colonic health including causing colorectal cancer, promoting tumorigenesis, and exacerbating ulcerative colitis [55]. Generally speaking, increased SCFAs and decreased pH, ammonia and BCFAs are considered beneficial colonic fermentation parameters [56].

Carbohydrates in soybean meal are predominantly non-starch polysaccharides (NSP) and free sugars (e.g., sucrose, stachyose and raffinose) [57]. The soybean NSPs can be further divided into insoluble cellulose, partially soluble non-cellulose polymers (e.g., mannans, galactans and fructans) and partially soluble pectic polysaccharides. The OSs in free sugars are indigestible but highly fermentable in monogastric animals [56,58]. However, cellulose in FPP has been deconstructed by manually added enzymes to release sugars for microbes to convert into proteins and exopolysaccharides [14]. Major soybean OSs (stachyose and raffinose) are also barely detectable in the FPP [17]. Compared to regular soybean meal, the FPP is supposed to have less fermentable carbohydrates. However, chitin is the major carbohydrate in fungus and is usually insoluble [59]. The carbohydrates from fungal biomass in the FPP may have played a role in the overall dietary fiber composition of the product.

In this study, the fecal pH surprisingly was maintained similar regardless of differences in ammonia and SCFAs concentrations in cats fed across the experimental diets. Such stable fecal pH and variable ammonia and total volatile fatty acids concentrations in dogs has been observed before [60]. This might be attributed to other fermentation compounds that also affect fecal pH such as lactic acid. Viscous guar gum shifted dietary amino acids from metabolic use to fermentation substrate in domestic cats [61]. Meanwhile, it has been reported that the microbe used in production of FPP can efficiently convert a broad range of difficult-to-metabolize oligosaccharides into cell mass and a microbial gum [14]. The linear increase in fecal ammonia concentration as FPP inclusion increased might be explained by fungal gum shifting amino acid to colonic fermentation to some extent. The increase in the isobutyrate/total fatty acids ratio and the isovalerate/total fatty acids ratio with an increase in FPP also supported this. As the ATTD of protein increased with FPP increase, it would be worthy to evaluate the in vivo apparent ileal digestibility of protein and amino acids of the FPP.

The decreased fecal SCFA content in cats fed with 5FPP and 10FPP compared to cats fed with SBM was expected as FPP had lower fermentable carbohydrates than soybean meal. However, this effect seemed to disappear when soybean meal was completely replaced by FPP in cat diets. The possible reasons remain to be investigated but might be related to a combined influence of carbohydrates and proteins on colonic microbiota composition and fermentation patterns [62]. It was also observed that fecal valerate concentration was comparable to, or higher than isobutyrate or isovalerate in cat feces in this study and some other cat studies [47,63,64] while it was the opposite in dogs [60,63,65,66]. While fecal valerate mostly comes from protein fermentation in the colon, an in vitro study using cheetah feces showed that some carbohydrate can also generate a small but similar amount of valerate and isobutyrate but less isovalerate [67]. Such discrepancies in fecal BCFAs between cats and dogs reflected possible differences in colonic fermentation characteristics such as microbiota profile, preference for substrate and epithelial absorption for fermentation products in the two species.

### 4.6. Palatability

The results indicated that relatively low inclusion of FPP contributed to palatability more than high inclusion of FPP. Such observation was consistent with previous studies that reported palatability of diets with yeast fermented grain products (CFP) were greater than control diets only when the inclusion level of CFP was lower (5 versus 10 and 15% or 150 versus 300 mg/kg BW) [40,47,68]. The FPP is also a fungal fermented grain product that might have common characteristics with yeast products that yielded such distinctive palatability pattern in cats. According to the amino acid profile of FPP reported in this study and that of soybean meal from 10 facilities in the U.S. reported by Grieshop et al. (2003) [69], the FPP had greater glutamic acids compared to soybean meal, which should have provided a stronger umami smell that is appealing to cats [70]. However, the reduced palatability at a high level of FPP or yeast fermentation products remained unclear. In a recent review, many studies have shown that cats are more selective towards food compared to dogs and are more prone to display neophobia for foods [71]. It has been reported that a higher inclusion level of *Saccharomyces cerevisiae* fermentation product may have resulted in overly strong and unfamiliar flavors and aromas that were questioned by cats [68]. However, cats in our digestibility study willingly consumed all experimental diets and no refusals were observed.

The palatability of FPP differed between dogs (unpublished data of the same authors) and cats as dogs preferred a 15% to lower FPP inclusion level in previous study while cats preferred a 10% to either lower or higher FPP inclusion level in this study. This may also be explained that cats are more sensitive to unfamiliar flavors than dogs are.

## 5. Conclusions

Replacement of soybean meal with FPP at up to 15% increased kibble expansion without significant changes in extrusion settings. The nutrient digestibility in cats of FPP-containing diets were comparable to that of soybean meal control diet. A generally decreased colonic fermentation was observed when FPP was included at 5–10% but not at 15% in cat diets, but all colonic fermentation parameters were within normal ranges. In combination with palatability test results, 10% seemed to be a favorable inclusion level of FPP in extruded cat diets. Further research on FPP carbohydrate composition and fermentability is needed to understand its influence on colonic fermentation related to the inclusion level.

## Figures and Tables

**Table 1:** Cat diet formulas with nutrient compositions of the experimental diets with increasing levels of FPP (SBM, 0%; 5FPP, 5%; 10FPP, 10%; and 15FPP, 15%).

Ingredient, %	SBM	5FPP	10FPP	15FPP
Soybean meal	15.00	10.00	5.00	0.00
FPP	0.00	5.00	10.00	15.00
Corn	38.26	37.97	40.06	42.16
Chicken meal	29.50	28.44	18.27	8.10
Chicken meal, low ash	1.83	3.18	11.25	19.32
Fish meal	3.00	3.00	3.00	3.00
Beet pulp	4.00	4.00	4.00	4.00
Salt	0.25	0.25	0.25	0.25
Titanium dioxide	0.40	0.40	0.40	0.40
Potassium chloride	0.25	0.25	0.25	0.25
Choline chloride, 60% dry	0.20	0.20	0.20	0.20
Natural antioxidant, dry	0.04	0.04	0.04	0.04
Natural antioxidant, liquid	0.03	0.03	0.03	0.03
Vitamin premix ^1^	0.15	0.15	0.15	0.15
Trace mineral premix ^2^	0.10	0.10	0.10	0.10
Chicken fat (topical)	6.00	6.00	6.00	6.00
Dry cat flavor (topical)	1.00	1.00	1.00	1.00
**Analyzed nutrient composition**				
Dry matter, %	95.72	94.60	94.06	93.73
Dry matter basis				
Crude protein, %	35.59	37.65	37.93	36.79
Crude fat, %	13.24	12.95	13.68	13.26
Ash, %	9.70	9.44	8.33	6.81
Total dietary fiber, %	20.16	16.38	15.84	16.22
Soluble dietary fiber, %	3.13	4.44	3.30	3.31
Insoluble dietary fiber, %	17.03	11.95	12.55	12.91
Goss energy, kcal/kg	4686.4	4809.9	4998.9	4950.9

^1^ Vitamin premix: 5.51% moisture, 4.02% crude protein, 34.5% ash, 13.4% calcium, 17,162,999 IU/kg vitamin A, 920,000 IU/kg vitamin D, 79,887 IU/kg vitamin E, 14,252 mg/kg thiamine, 4719 mg/kg riboflavin, 12,186 mg/kg pantothenic acid, 64,736 mg/kg niacin, 5537 mg/kg pyridoxine, 720 mg/kg folic acid, 70 mg/kg biotin, 22 mg/kg vitamin B_12_. ^2^ Trace mineral premix: 0.66% moisture, 21.5% calcium, 0.02% sodium, 0.57% magnesium, 38,910 mg/kg iron, 11,234 mg/kg copper, 5842 mg/kg manganese, 88,000 mg/kg zinc, 1584 mg/kg iodine, 310 mg/kg selenium, 19% carbohydrate, and 1% crude fat. FPP, fermented plant protein.

**Table 2:** Content of essential amino acids in FPP and its and amino acid score for adult cats at maintenance.

Amino Acid	FPP, mg/g Crude Protein	NRC References ^1^, mg/g Crude Protein	AAS
Arginine	66.06	38.5	1.72
Histidine	26.40	13.0	2.03
Isoleucine	50.60	21.5	2.35
Leucine	79.70	51.0	1.56
Lysine	58.70	17.0	3.45
Phenylalanine	53.15	22.0	2.42
Phenylalanine + tyrosine	83.46	76.5	1.09
Methionine	13.73	8.5	1.62
Methionine + cystine	27.59	11.3	2.43
Threonine	38.60	26.0	1.48
Tryptophan	17.40	6.5	2.68
Valine	50.30	25.5	2.00
**PDCAAS calculation**			
Limiting amino acid	Methionine and SAA		
In vitro protein digestibility, %	98.46		
PDCAAS	1.07		

^1^ The essential amino acid recommended allowances of adult cats at maintenance according to the National Research Council [19]. Used as the reference amino acid pattern to calculate AAS. FPP—fermented plant protein; AAS—amino acid score; SAA, sulfur amino acids (methionine and cystine); PDCAAS—protein digestibility corrected amino acid score.

**Table 3:** Least square means and contrasts (SBM vs. 5–15FPP [*T*]; linear [*L*]; quadratic [*Q*]; cubic [*C*]) for extrusion processing data and post-extrusion kibble measurements of experimental diets.

	Treatment ^1^					*p*-Value			
Parameter	SBM	5FPP	10FPP	15FPP	MSE	SBM vs. *T*	*L*	*Q*	*C*
Sample size	5	5	7	4					
Feed rate, kg/h	597.80	595.00	597.57	596.75	2.44	0.3645	0.8194	0.1140	0.2991
PC steam, kg/h	29.60	29.20	29.57	30.00	0.47	0.8967	0.4276	0.9461	0.0240
Motor load, %	73.00	72.40	70.57	70.75	2.80	0.2713	0.1084	0.8680	0.6733
Motor power, kW	31.82	32.00	30.94	30.93	1.65	0.4163	0.2496	0.9136	0.5072
SME, kJ/kg	53.13	53.71	51.70	51.77	2.79	0.3762	0.2496	0.7956	0.4627
EX water, kg/h	17.20	17.20	17.29	17.50	0.49	0.9503	0.5508	0.6274	0.4358
Knife speed, rpm	1500 ^a^	1500 ^a^	1378 ^b^	1406 ^b^	22.37	<0.001	<0.001	0.1825	0.0033
OE Bulk density, kg/m^3^	421.60	416.40	411.00	410.50	12.26	0.3762	0.1204	0.6083	0.7856
Sample size	50	50	70	40					
Diameter, mm	7.91 ^c^	8.25 ^b^	8.39 ^a^	8.30 ^ab^	0.27	<0.0001	<0.0001	0.0012	0.0708
Thickness, mm	4.71 ^b^	4.57 ^c^	4.88 ^a^	4.86 ^a^	0.27	<0.0001	<0.0001	0.0307	0.001
SEI	2.50 ^c^	2.72 ^b^	2.76 ^ab^	2.82 ^a^	0.17	<0.0001	<0.0001	0.0016	0.0798

^1^ Control (SBM), no fermented plant protein added; and 5%, 10% and 15% (5FPP, 10FPP and 15FPP) fermented plant protein added to offset soybean meal in the diet, respectively. ^abc^ Means within a row with different superscripts differ (*p* < 0.05). PC—preconditioner; EX-extruder; OE-off the extruder.

**Table 4:** Least square means and contrasts (SBM vs. 5–15FPP [*T*]; linear [*L*]; quadratic [*Q*]; cubic [*C*]) for food intake, fecal output, fecal score, and defecation frequency of cats fed diets containing increasing levels of FPP.

	Treatment ^1^					*p*-Value			
Parameter	SBM	5FPP	10FPP	15FPP	SEM	SBM vs. *T*	*L*	*Q*	*C*
Intake (DM), g/d	79.13	77.32	76.13	77.37	3.875	0.1338	0.0585	0.7835	0.4936
Fecal output (As-is), g/d	58.10	52.14	53.25	54.23	4.543	0.5903	0.2737	0.3269	0.3292
Fecal output (DM), g/d	18.68	16.99	17.41	16.52	1.349	0.9843	0.2277	0.1053	0.9707
Fecal moisture, %	67.70 ^b^	67.04 ^b^	67.12 ^b^	69.78 ^a^	0.016	0.0241	0.5603	0.0139	<0.001
Fecal score ^2^	3.86	3.84	3.86	3.82	0.083	0.7502	0.8413	0.4871	0.7836
Defecation time/day	1.10	1.05	1.00	1.08	0.091	0.3043	0.3632	0.7979	0.4933

^1^ Control (SBM), no fermented plant protein added; and 5%, 10% and 15% (5FPP, 10FPP and 15FPP) fermented plant protein added to offset soybean meal in the diet, respectively. ^2^ Subjective 1 to 5 scale with 1, runny; 2, soft; 3, firm and moist; 4, firm; 5, dry and hard [20]. ^ab^ Within a row, means without a common superscript differ (*p* < 0.05).

**Table 5:** Least square means and contrasts (SBM vs. 5–15FPP [*T*]; linear [*L*]; quadratic [*Q*]; cubic [*C*]) for the nutrient ATTD calculated using the titanium dioxide marker method by cats fed diets with increasing levels (0%, 5%, 10%, and 15%) of FPP.

	Treatment ^1^					*p*-Value			
Parameter	SBM	5FPP	10FPP	15FPP	SEM	SBM vs. *T*	*L*	*Q*	*C*
Total fecal collection method									
Dry matter, %	78.23	79.66	78.73	80.57	0.009	0.4613	0.5432	0.0716	0.5745
Organic matter, %	84.65	85.60	84.54	84.97	0.010	0.4511	0.7269	0.2631	0.5203
Crude protein, %	75.71	78.21	78.07	80.11	0.017	0.9613	0.0802	0.0499	0.5049
Gross energy, %	82.91	84.20	85.53	83.83	0.007	0.0274	0.0239	0.7712	0.2474
Crude Fat, %	93.48 ^ab^	92.17 ^b^	94.56 ^a^	94.98 ^a^	0.007	0.0969	0.0131	0.3927	0.0031
TiO_2_ marker method									
Dry matter, %	70.33 ^ab^	72.75 ^a^	68.33 ^b^	73.08 ^a^	0.010	0.0018	0.1913	0.0006	0.4850
Organic matter, %	78.83 ^ab^	80.67 ^a^	77.25 ^b^	79.33 ^ab^	0.015	0.0045	0.0502	0.0063	0.4239
Crude protein, %	67.33 ^b^	70.92 ^ab^	67.75 ^b^	72.50 ^a^	0.017	0.0632	0.5766	0.0008	0.3947
Gross energy, %	78.37	80.27	77.72	79.31	0.007	0.0471	0.3358	0.0139	0.4594
Crude Fat, %	91.33 ^ab^	89.67 ^b^	92.17 ^a^	93.08 ^a^	0.008	0.2347	0.0286	0.5194	0.0010

^1^ Control (SBM) no fermented plant protein added, and 5%, 10% and 15% (5FPP, 10FPP and 15FPP) fermented plant protein added to offset soybean meal in the diet, respectively. ^ab^ Within a row, means without a common superscript differ (*p* < 0.05).

**Table 6:** Least square means and contrasts (SBM vs. FPP10–30 [*T*]; linear [*L*]; quadratic [*Q*]; cubic [*C*]) for short-chain fatty acid (SCFA), branched-chain fatty acid (BCFA), and total fatty acids (SCFAs + BCFAs) production from the fresh fecal sample collected from the cats fed diets with increasing levels (0%, 5%, 10%, and 15%) of FPP expressed in a mmol/g of feces in dry matter (DM) basis.

Parameter	Treatment ^1^					*p*-Value			
	SBM	5FPP	10FPP	15FPP	SEM	SBM vs. *T*	*L*	*Q*	*C*
Fecal pH	5.73	5.73	5.74	5.53	0.100	0.2358	0.4973	0.0608	0.0233
Ammonia, μmol/g DM feces	102.5 ^b^	122.0 ^ab^	127.0 ^ab^	139.8 ^a^	12.4	0.0498 ^2^	0.0434	0.1059	0.5142
SCFA, μmol/g DM feces	452.8 ^a^	392.1 ^ab^	384.9 ^b^	456.1 ^a^	34.81	0.0200	0.0793	0.7617	0.0020
Acetate	278.5 ^ab^	245.9 ^ab^	241.4 ^b^	283.6 ^a^	20.97	0.0290	0.1291	0.6517	0.0034
Propionate	125.3 ^ab^	104.5 ^b^	108.3 ^ab^	127.7 ^a^	11.34	0.0388 ^2^	0.2933	0.9015	0.0022
Butyrate	49.0	41.7	35.3	44.9	5.77	0.0505	0.0527	0.7879	0.2274
BCFA, μmol/g DM feces	27.35	22.36	22.22	27.53	4.710	0.1494	0.2766	0.9394	0.0331
Isobutyrate	5.98	6.05	6.10	6.94	1.42	0.5220	0.3571	0.1376	0.0666
Isovalerate	5.65	6.41	6.13	7.07	0.751	0.6154	0.2698	0.0517	0.4255
Valerate	15.72	9.91	9.98	13.52	3.049	0.1334	0.0879	0.5178	0.0402
Isobutyrate/total FA ratio	1.23	1.44	1.44	1.48	0.002	0.2176	0.0347	0.2727	0.4665
Isovalerate/total FA ratio	1.16 ^b^	1.58 ^a^	1.53 ^ab^	1.49 ^ab^	0.001	0.0235 ^2^	0.0258	0.0575	0.1662
Total FA, μmol/g DM feces	480.1 ^ab^	414.4 ^bc^	407.1 ^c^	483.6 ^a^	38.86	0.0170	0.0712	0.7651	0.0015

^1^ Control (SBM) no fermented plant protein added, and 5%, 10% and 15% (5FPP, 10FPP and 15FPP) fermented plant protein added to offset soybean meal in the diet, respectively. ^2^ *p* value from Tukey’s post hoc pair comparison. ^abc^ Within a row, means without a common superscript differ (*p* < 0.05). FA, fatty acids.

**Table 7:** Palatability assessment of diets containing FPP (diet B) relative to the control (SBM, diet A) by 30 cats in a two-day split-bowl trial.

Diet A vs. B	First Choice of Diet A, n ^1^		Intake Ratio of Diet A ^2^	
	Day 1	Day 2	Day 1	Day 2
SBM vs. 5FPP	24 *	19	0.541	0.483
SBM vs. 10FPP	18	14	0.342 *	0.296 *
SBM vs. 15FPP	13	17	0.423	0.410 *

^1^ First choice number of first visits to bowl with diet B can be obtained by 30 − n. ^2^ Intake ratio of diet A = average of intake (g) of diet A/total intake (g) of diets A + B. * Means data are different between the two diets (*p* < 0.05).

## Data Availability

Data are contained within the article. Further inquiries can be directed to the corresponding author.

## Abbreviations

The following abbreviations are used in this manuscript:

FPP	Fermented plant protein
TiO_2_	Titanium dioxide
ATTD	apparent total tract digestibility
PC	Preconditioner
SME	specific mechanical energy
SEI	Sectional expansion index
ME	Metabolizable energy
CF	Crude fat
CP	Crude protein
NFE	Nitrogen-free extract
DM	Dry matter
OM	Organic matter
TDF	Total dietary fiber
IDF	Insoluble dietary fiber
SDF	Soluble dietary fiber
GE	Gross energy
SCFA	Short chain fatty acids
TFC	Total fecal collection
